# Diagnosis of propofol allergy (the innovative role of the basophil activation test in the identification of allergen): a case report

**DOI:** 10.11604/pamj.2025.50.82.46400

**Published:** 2025-03-21

**Authors:** Chaimaa Chahine, Zakaria Zidane, Karima Mohtadi, Sanaa Souat, Khadija El azhary, Rachid Saïle, Abdallah Badou, Mohammed Ali Debbarh, Claude Lambert, Ibtihal Benhsaien, Hanane Salih Alj

**Affiliations:** 1 Laboratory of Biology and Health, Research Center of Biotechnologies and Health, Faculty of Sciences Ben Msik, University Hassan II of Casablanca, Avenue Cdt Driss El Harti, Sidi Othmane, Casablanca, Morocco,; 2 Laboratory of Immunogenetics and Human Pathology, University Hassan II of Casablanca, Faculty of Medicine and Pharmacy, 19 Rue Tarik Ibnou Ziad, Casablanca, Morocco,; 3 Hepato-Gastroenterology, Hassan II University of Casablanca, Casablanca, Morocco,; 4 Laboratory of Immunology, CNRS UMR 5307 Labo Georges Friedel, Biology-Pathology Center, St Etienne, France,; 5 Laboratory of Clinical Immunology, Inflammation and Allergies, University Hassan II of Casablanca, Faculty of Medicine and Pharmacy, 19 Rue Tarik Ibnou Ziad, Casablanca, Morocco

**Keywords:** Propofol allergy, basophil activation test (BAT), anaphylaxis, hypersensitivity reactions, case report

## Abstract

Drug allergies (DA), particularly to anesthetics, are underexplored in Morocco. Traditional diagnostic methods such as skin tests and specific IgE assays are costly, risky, and often inaccessible. The basophil activation test (BAT) is emerging as a safer and more effective alternative, especially for diagnosing propofol allergy, an under-researched area. This study examines the role of the basophils activation test in diagnosing perioperative anaphylaxis to propofol. A 32-year-old woman experienced two anaphylactic reactions during fibroscopy after propofol administration. The basophil activation test (BAT) was performed one week later using diluted propofol, measuring basophil activation via CD63 and CD203c markers by flow cytometry. Results showed 0% basophil activation in the negative control, 85.7% in the positive control, and 19.05% at 1 mg/ml of propofol, with a strong correlation (0.973) between concentration and activation. The basophil activation test (BAT) offers a reliable diagnostic method, particularly in Morocco, where traditional tests are limited, providing a cost-effective solution for diverse allergens in low-resource settings.

## Introduction

Drug allergy (DA), particularly anesthetic allergy, remains a relatively unexplored field in Morocco. Skin tests and specific IgE assays, although used to diagnose allergies, reveal certain sensitization and demonstrate clinical relevance, especially for specific IgEs. These tests are only available for a limited number of drugs and are rarely performed for drug allergy diagnosis in Morocco due to their high cost [1]. Indeed, for most patients, these tests remain expensive and carry significant risks, potentially leading to severe allergic reactions. Allergic events related to anesthetics, though rare, can be life-threatening, with an estimated incidence ranging from 1 in 3,500 to 1 in 20,000 surgical interventions, and a mortality rate varying between 3% and 9% [2]. In such circumstances, any perioperative event that suggests an allergic reaction should be followed by a thorough allergological assessment to identify the responsible drugs and prevent anaphylactic episodes during future anesthesia. This approach is even more challenging in Morocco, where specific IgE tests are not available, and anesthetic allergy consultations are not yet established. The basophil activation test (BAT) has emerged as a promising alternative for the diagnosis of drug allergies. This in vitro test assesses basophil degranulation induced by allergen contact in the presence of IgE, offering a safer method than skin tests [3]. Positive BAT results have been reported for several drug classes, including antibiotics, muscle relaxants, hypnotics, and analgesics. However, its specific effectiveness for propofol, a hypnotic commonly used in general anesthesia, remains to be confirmed due to the limited number of available studies. In a 2017 study, the authors showed that three patients had positive BAT results: one for metamizole, one for PPL (benzylpenicillin polylysine), and one for pancuronium, while BAT results for propofol were inconclusive [3]. This study aims to investigate BAT's role in diagnosing propofol allergy after suspected perioperative anaphylaxis, addressing limitations of traditional tests and improving patient safety during anesthesia, especially in low-resource settings like Morocco.

## Patient and observation

**Patient information:** a 32-year-old female patient experienced two anaphylactic shocks during a gastroscopy performed for gastrointestinal diagnosis, her family history includes a grandmother who suffered from a severe allergy to shellfish, which triggered anaphylactic shock. Her medical history reveals allergic asthma triggered by dust mites since the age of one, with symptoms still present but well-controlled by treatment. The allergological anamnesis indicates a previous anaphylactic shock, including respiratory difficulties and swelling of the face and throat, after ingesting shellfish or inhaling their volatile proteins. Additionally, she developed urticaria after consuming peanuts and eggs, leading her to avoid these foods to prevent further allergic reactions. Due to the high risks and costs of allergy testing, the patient did not consult an allergist despite a severe history of hypersensitivity to propofol during two anesthetic procedures. To ensure safety, the basophil activation test (BAT) was performed as a first-line diagnostic tool, offering a lower-risk alternative to confirm the allergy and prevent life-threatening reactions during future anesthesia.

**Clinical findings:** both anaphylactic episodes were marked by immediate hypersensitivity reactions shortly after awakening from anesthesia with propofol. Physical findings included skin redness, urticaria, and respiratory compromise.

**Timeline of current episode:** July 2021: an immediate hypersensitivity reaction occurred a few minutes after a gastroscopy, following the injection of 200 mg of propofol alone (Diprivan®). Upon awakening, the patient presented with pruritus on the extremities, urticaria on the upper limbs, bronchospasm, and desaturation. Her condition improved rapidly after the administration of oxygen, along with an injection of 0.1 mg of adrenaline and 120 mg of corticosteroids (Solumédrol®). After this reaction, she did not follow up with an allergist or undergo any allergy testing. ii)July 2024: during a subsequent gastroscopy, the patient again received only propofol (Diprivan®) as an anesthetic. Shortly after awakening, she experienced another immediate hypersensitivity reaction, characterized by urticaria on the upper limbs, skin redness, pruritus on the extremities, tachycardia (118 bpm), hypotension (90/60 mm Hg), and bronchospasm. The treatment included an injection of 120 mg of corticosteroids (Solumédrol®), 10 mg of antihistamine (Zyrtec®), and Ventoline® to relieve the symptoms.

**Figure 1:**
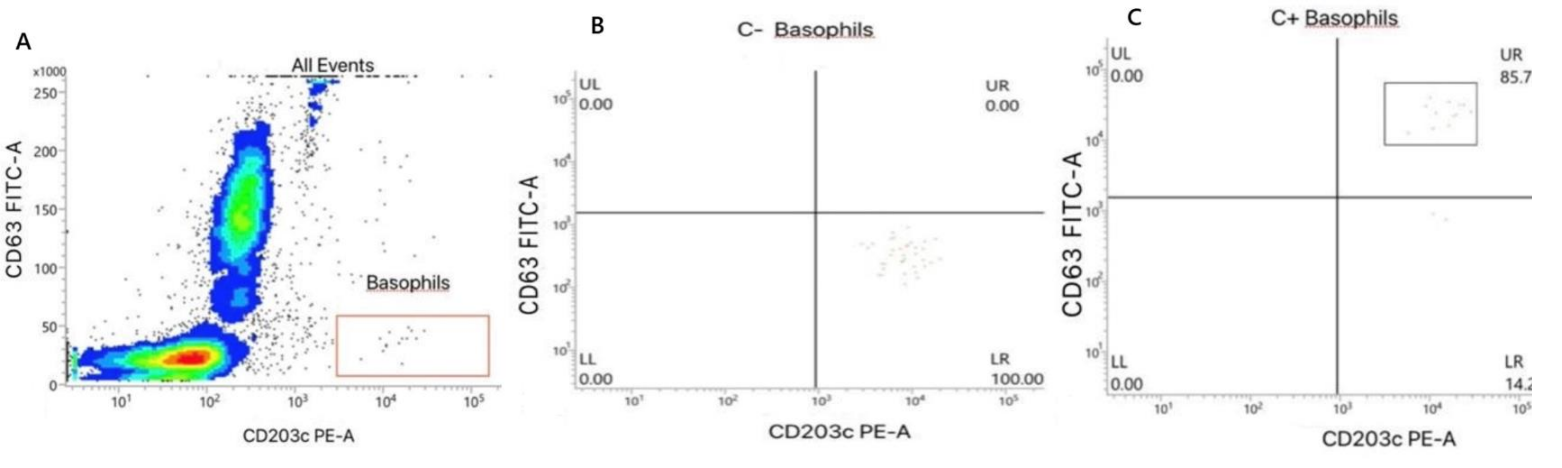
strategy for identifying activated basophils A) identification of basophils among all blood cells; B) negative control; C) positive control; CD63: degranulation marker; FITC (fluorescein Isothiocyanate): fluorochrome emitting green in cytometry (FITC channel-A) and CD203c: activation marker; PE (phycoerythrin): fluorochrome emitting orange-red in cytometry (PE channel-A); CD203c PE-A and CD63 FITC-A have a logarithmic scale ranging from approximately 10^0^(1) to 10^5^(100,000)

**Diagnostic assessment:** to avoid risks associated with skin testing, the basophil activation test (BAT) was performed one week after the second episode. Propofol (Diprivan® 10 mg/ml, Corden Pharma S.P.A, Italy) was tested at dilutions of 1/10 (1 mg/ml), 1/50 (0.2 mg/ml), and 1/100 (0.1 mg/ml) diluted in Phosphate-Buffered Saline (PBS). The blood sample was collected in a heparinized tube. The test was performed on whole blood within 4 hours of collection. Basophil activation was measured via CD63 and CD203c+ markers using the BasoFlowEx® kit (EXBIO, Czech Republic). Briefly, 100 µl of whole blood and 100 µl of the stimulation buffer provided in the kit were added to all tubes, gently vortexed, and pre-incubated at 37°C for 25 minutes in an air incubator. Then, 10µl of allergen solution, diluted to 1/10, 1/50, and 1/100, was added to the sample. For the positive control, 10µl of a stimulation control, including an anti-IgE antibody and N-formyl-Met-Leu-Phe (fMLP), were added. For the negative control, 10µl of PBS were added. The tubes were then vortexed and incubated at 37°C for 25 minutes in an air incubator, followed by mixing with 20 µl of labeling reagent containing anti-CD63 FITC and anti-CD203c PE antibodies. After an additional 20-minute incubation at +4°C, 300µl of lysing solution were added for 5 minutes at room temperature, followed by 4 ml of deionized water for 10 minutes. After centrifugation at 300x g for 5 minutes, the supernatant was removed, and the cell pellet was resuspended in 0.4 ml of PBS + 2% Fetal Bovine Serum (FBS). The samples were analyzed using a flow cytometer (BD FACS Lyric, Becton Dickinson and Company, USA). To analyze a sufficient number of basophils (>200), the acquisition of 100,000 events (CD203c+, SSC low) was set as the gating strategy. Basophil identification among the total blood cells and activated CD63+ basophils was determined as shown in Figure 1A. The threshold for determining a positive test was set at 5% CD63+ basophils, following the manufacturer's instructions. The level of basophil activation was expressed as the percentage of CD63+ basophils above the threshold defined in the negative control.

**Diagnosis:** the elevated activation markers (CD63 and CD203c) in response to propofol at all dilutions tested indicate a clear hypersensitivity reaction. The patient's history of immediate allergic symptoms (urticaria and anaphylactic shock) is consistent with these findings, reinforcing the diagnosis of propofol allergy.

**Therapeutic interventions:** emergency treatments were provided during each reaction, including adrenaline, corticosteroids, antihistamines, and bronchodilators. During the first episode of anaphilaxis in July 2021, there was administration of oxygen, as well as an injection of 0.1 mg of adrenaline and 120 mg of corticosteroids (Solumédrol®). During the second episode of anaphilaxis in July 2024, the treatment included an injection of 120 mg of corticosteroids (Solumédrol®), 10 mg of antihistamine (Zyrtec®) and Ventoline® to relieve symptoms. Basophil activation test was utilized to confirm hypersensitivity while avoiding further allergenic exposure.

**Figure 2:**
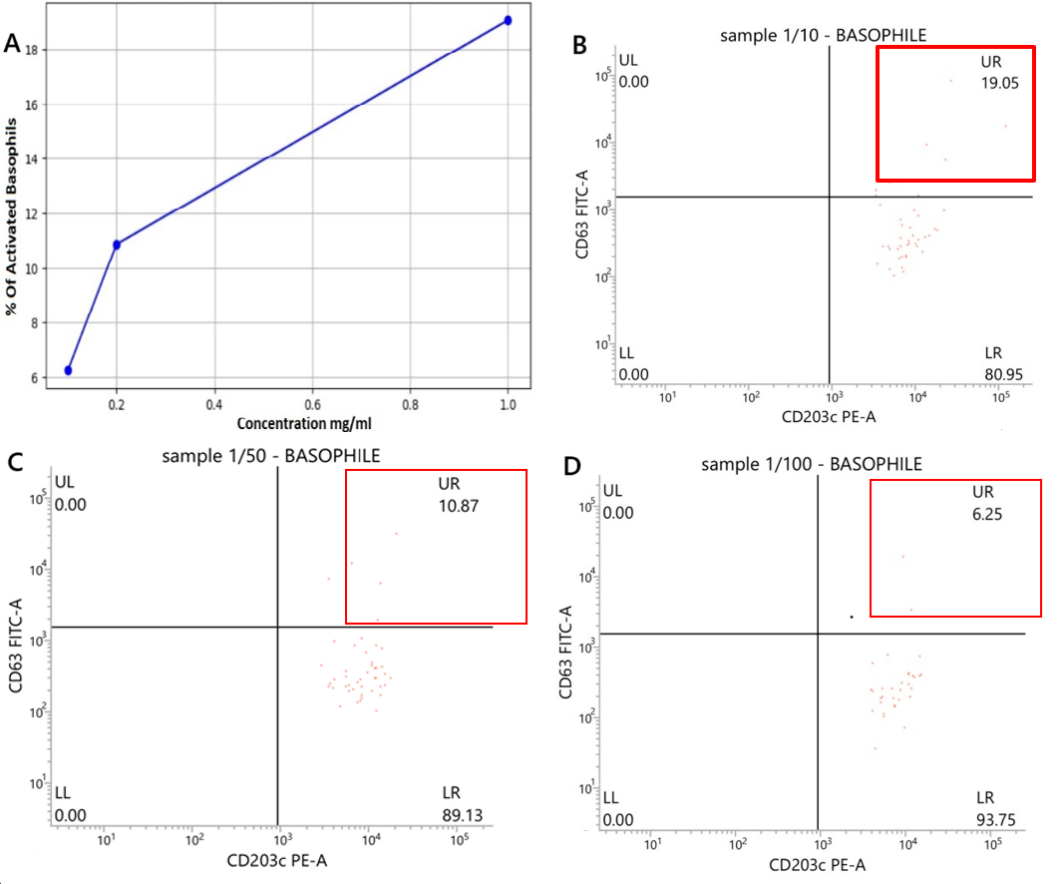
basophil activation test with propofol A) Percentage of activation as a function of concentration; B, C, D) percentage of CD63 expression by basophils in response to different doses of propofol (D1= 1/10, D2= 1/50, D3= 1/100); CD63: degranulation marker; FITC (fluorescein Isothiocyanate): fluorochrome emitting green in cytometry (FITC Channel-A) and CD203c: activation marker; PE (phycoerythrin): fluorochrome emitting orange-red in cytometry (PE channel-A); CD203c PE-A and CD63 FITC-A have a logarithmic scale ranging from approximately 10^0^ (1) to 10^5^

**Follow-up and outcomes:** basophil activation test confirmed the diagnosis, enabling avoidance of propofol in future anesthetic plans. No adverse events occurred during the test. In the negative control, 0% (Figure 1B) of basophils were activated, indicating that the basophils were at rest in the absence of any stimulus. In contrast, the positive control showed 85.7% of basophils activated (Figure 1C). Basophil activation decreases with drug dilution but remains detectable (Figure 2A), with a correlation coefficient of 0.973 and an associated p-value of 0.027. Drug exposure led to 19.05% basophil degranulation at 1 mg/ml (Figure 2B). This reaction correlated with drug concentration, with degranulation rates of 10.87% at 0.2 mg/ml (Figure 2C) and 6.25% at 0.1 mg/ml (Figure 2D), these values remaining above the 5% threshold, although significantly lower than those observed at higher concentrations. Statistical analyses were performed using Python software to study the relationship between drug concentration and the percentage of basophil activation caused by propofol, as well as Pearson's correlation coefficient.

**Patient perspective:** as a patient, I felt an immense sense of relief when the cause of my severe reactions was identified. Not knowing the trigger had been a constant source of anxiety for me. I was particularly grateful for the non-invasive nature of the BAT, as it provided clarity without exposing me to further risks. This diagnosis has given me confidence and peace of mind for any future medical procedures."

**Informed consent:** this study was conducted in accordance with the principles outlined in the Helsinki Declaration and obtained approval from the regional ethics committee “Medecine and pharmacy faculty of Casablanca/university hospital center bnou Rochd Casablanca” N° ordre: 11/2022, to ensure that the study complied with ethical standards and protected the rights and well-being of the participants (Law 28-13, N° 02/DRC/00). Before participating in the study, all legal guardians of participants were required to provide informed consent. Confidentiality of the data collected was ensured by using identifiers rather than the names of participants. The patient provided verbal and written consent for the BAT and agreed to the publication of her anonymized case details for educational purposes.

## Discussion

Several anesthetic agents are used during general anesthesia, most of which tend to cause allergic reactions and anaphylaxis. During anesthesia, patients are exposed to numerous drugs and other non-medicinal substances over a relatively short period [3]. Anaphylaxis due to propofol is very rare, and propofol (2,6 di-isopropyl phenol) is commonly used today as an induction agent. Anaphylaxis to propofol occurs in 1.2% of cases of perioperative anaphylactic shock, with skin tests to reliably predict this reaction, unlike many other drugs [2,4]. In this clinical case the BAT results show positive results, the concentration of (1 mg/ml) from the stock solution, shows significant basophil activation (19.05%), some types of anesthetics in the range of 5 µg/ml to 500 µg/ml can also cause basophil activation and induce allergic reactivity in a BAT [5]. A study conducted in 2017 by Hoffman *et al*. found significant basophil activation at propofol concentrations similar to those tested, with dose-dependent response levels [6]. The strong correlation between allergen concentration and basophil response reflects the upregulation of CD63, a sign of basophil activation that is specifically linked to FcÎµRI receptor stimulation. In our study, BAT results were obtained one week after the propofol reaction. The recommendation is to do this 4 to 6 weeks after the accident to avoid a refractory state of the basophils. However, it is not always possible to wait before reanesthetizing to complete the surgical procedure, and our results show that basophil reactivity is well detectable even after only one week their reactivity is a key indicator of allergic sensitivity [6,7]. Generally speaking, after an allergic reaction, basophil reactivity can be reduced over time by treatment and removal of the allergen.

That's why it's important to identify the allergy after a surgical accident and not wait for another need for intervention, which may also be urgent and not allow time for allergological testing. There are concerns that the emulsified preparation of Diprivan®, which consists of propofol, an alkylphenol derivative (2,6 di-isopropyl phenol) as well as 10% 2.25% glycerol, soybean oil, and 1.2% egg lecithin, may not be safe for patients allergic to eggs, peanuts, and soy, due to the phenomenon of dietary cross-reactivity [7]. In our case, the work-up and allergological tests were not carried out, and according to the patient's history, she has suffered from asthma since the age of one and has had urticaria after ingesting egg and peanut-containing foods, and is therefore possibly sensitized to proteins of the PR-10 family [8]. Researchers have reported cases of hypersensitivity reactions to propofol, particularly in peanut-allergic patients, whose worldwide prevalence is estimated at between 0.6% and 3% [9]. A recent study in Morocco identified peanuts as the third most common food allergen in the country, and allergic reactions linked to peanut oil, used in some pharmaceutical preparations, have also been reported [1]. In addition, because they belong to the same botanical family, peanuts and soya can cause cross-reactions, with soya often being responsible for anaphylaxis in adults. Nevertheless, several studies have shown contradictory results regarding the use of this drug in people with soy and egg allergies, and this issue remains unresolved [10]. Indeed, the two isopropyl groups of propofol could be the two epitopes making propofol a divalent molecule capable of bridging the two specific IgE receptors, allowing them to bind to several IgE receptors on immune cells [8,10].

These reactions occur on re-exposure to propofol, when IgE antibodies bind to mast cells and basophils, triggering their activation and causing an allergic reaction [2,10]. The BAT is a useful diagnostic tool for detecting drug allergy (DA), particularly when skin tests or specific IgE assays are not feasible. Although some studies suggest that BAT may have good sensitivity for identifying hypersensitivity to propofol, the available data are still limited and based on small samples, restricting the generalizability of results [2,3,10]. The accuracy of the BAT in this case is supported by the correlation between the test results and the clinical reactions of the patient, who had anaphylactic reactions only after the administration of propofol alone, without other drugs. To assess specificity, the absence of basophil activation in the negative control and high activation in the positive control is crucial, indicating that the BAT responds specifically, to propofol, thus reducing the risk of false positives. Larger studies are nevertheless needed to confirm these observations [10]. The BAT is a cost-effective diagnostic method for drug allergy (DA) in Morocco. At 6,000 MAD for a kit capable of performing up to 100 tests, or assessing 100 allergens, TAB is more cost-effective than specific IgE tests, which cost around 250 MAD per allergen. What's more, BAT can assess a wide range of allergens, including those for which specific IgE tests are not available, making it a versatile and valuable tool for allergy diagnosis. On the other hand, specific IgE tests are limited to a restricted number of drug allergens, which may diminish their effectiveness in certain clinical situations.

## Conclusion

The BAT therefore appears to us as a promising tool for guiding the management of DA and improving patient safety during anesthesia, particularly in our country where skin tests and specific IgE assays are expensive and not very accessible, have major limitations and are not always feasible.
